# An Updated Review of Genetic Associations With Severe Adverse Drug Reactions: Translation and Implementation of Pharmacogenomic Testing in Clinical Practice

**DOI:** 10.3389/fphar.2022.886377

**Published:** 2022-04-25

**Authors:** Chuang-Wei Wang, Ivan Arni C. Preclaro, Wei-Hsiang Lin, Wen-Hung Chung

**Affiliations:** ^1^ Department of Dermatology, Drug Hypersensitivity Clinical and Research Center, Chang Gung Memorial Hospital, Taipei and Keelung, Taiwan; ^2^ Cancer Vaccine and Immune Cell Therapy Core Laboratory, Chang Gung Memorial Hospital, Linkou, Taiwan; ^3^ Chang Gung Immunology Consortium, Chang Gung Memorial Cital and Chang Gung University, Taoyuan, Taiwan; ^4^ Department of Dermatology, Xiamen Chang Gung Hospital, Xiamen, China; ^5^ College of Medicine, Chang Gung University, Taoyuan, Taiwan; ^6^ Whole-Genome Research Core Laboratory of Human Diseases, Chang Gung Memorial Hospital, Keelung, Taiwan; ^7^ Department of Dermatology, Beijing Tsinghua Chang Gung Hospital, School of Clinical Medicine, Tsinghua University, Beijing, China; ^8^ Department of Dermatology, Ruijin Hospital, School of Medicine, Shanghai Jiao Tong University, Shanghai, China; ^9^ Genomic Medicine Core Laboratory, Chang Gung Memorial Hospital, Linkou, Taiwan

**Keywords:** adverse drug reactions, drug-induced liver injury, CYP, human leukocyte antigens, drug transporter, stevens-johnson syndrome, toxic epidermal necrolysis

## Abstract

Adverse drug reactions (ADR) remain the major problems in healthcare. Most severe ADR are unpredictable, dose-independent and termed as type B idiosyncratic reactions. Recent pharmacogenomic studies have demonstrated the strong associations between severe ADR and genetic markers, including specific HLA alleles (e.g., HLA-B*15:02/HLA-B*57:01/HLA-A*31:01 for carbamazepine-induced severe cutaneous adverse drug reactions [SCAR], HLA-B*58:01 for allopurinol-SCAR, HLA-B*57:01 for abacavir-hypersensitivity, HLA-B*13:01 for dapsone/co-trimoxazole-induced SCAR, and HLA-A*33:01 for terbinafine-induced liver injury), drug metabolism enzymes (such as CYP2C9*3 for phenytoin-induced SCAR and missense variant of *TPMT*/*NUDT15* for thiopurine-induced leukopenia), drug transporters (e.g., SLCO1B1 polymorphism for statin-induced myopathy), and T cell receptors (Sulfanilamide binding into the CDR3/Vα of the TCR 1.3). This mini review article aims to summarize the current knowledge of pharmacogenomics of severe ADR, and the potentially clinical use of these genetic markers for avoidance of ADR.

## Introduction

Adverse drug reaction (ADR) remains one of the leading causes of death around the world (Shoshi et al., 2015). More than 100,000 people have been reported to die by ADR every year (Alomar, 2014), and most severe ADR belongs to type B unpredictable reactions, which are rare, no connection to the dosage, and occur in individuals with an underlying genetic predisposition (Pirmohamed et al., 2004; Uetrecht, 2007). Type B ADR can be presented as skin injury and liver injury. Skin injury is classified from mild maculopapular exanthema (MPE) to life-threatening severe cutaneous adverse drug reactions (SCAR), including drug reactions with eosinophilia and systemic symptoms (DRESS), Stevens–Johnson syndrome (SJS), and toxic epidermal necrolysis (TEN). Although SCAR are rare, they affect approximately 2% of all hospitalized patients (Valeyrie-Allanore et al., 2007), with an incidence between 2 and 7 cases of SJS/TEN cases/million/per year (Mockenhaupt et al., 2008; Levi et al., 2009; Sassolas et al., 2010; Sekula et al., 2013) and 1/1,000 to 1/10,000 cases of DRESS (Amante et al., 2009). The mortality of DRESS, SJS, and TEN are approximately 2%, 1∼10%, and > 30%, respectively (Roujeau and Stern, 1994; Kardaun et al., 2013; Chung et al., 2016a; Mockenhaupt, 2017; Wang et al., 2018; Tsai et al., 2019). Furthermore, ADR also identified to induce hepatic toxicity, called as drug-induced liver injury (DILI). Approximately 10% of DILI patients may progress to acute liver failure (Yip et al., 2015), and the mortality of DILI is up to 7% (Björnsson and Björnsson, 2017). The incidence of DILI is estimated to be 1 to 10 per 100,000 new users (Yip et al., 2015). Since severe ADR can abe easily confused with other aetiologies of liver damage or renal impairment, the diagnosis of “drug-induced” and culprit drug are sometime difficult to determine. DILI can be further categorized into two classes, allergic and non-allergic. Allergic DILI is often related to HLA genetic factor and results in abnormal immune response; non-allergic DILI, on the other hand, is mostly the result of accumulation of related reagents within liver (Kuna et al., 2018).

## Genetic Factors of Severe Adverse Drug Reactions

**FIGURE 1:**
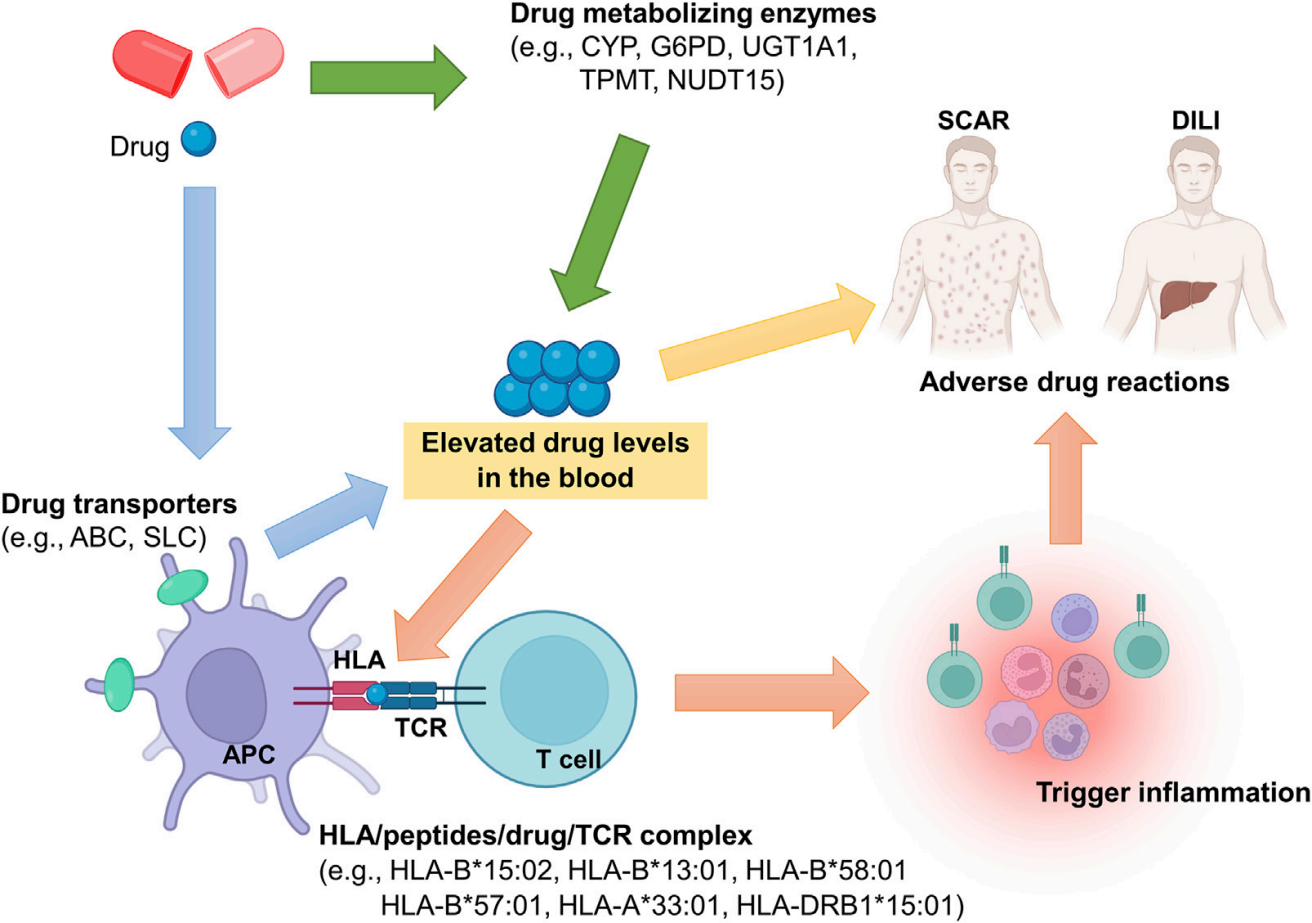
Potential genetic determinants involved in pathogenesis of severe ADR. Genetic polymorphisms in drug metabolizing enzymes or drug transporters may alter their function, and then elevated drug levels in the blood, resulting in ADR occurrence. Also, the drug may trigger immune responses through HLA/drug/TCR complex. In the HLA/drug/TCR model, HLA is considered as the key molecular for induction of ADR. Taken together, genetic polymorphisms of HLA, drug metabolizing enzyme, drug transporter, and TCR play important roles in ADR pathogenesis.

In this review, we summarize the currently identified genetic biomarkers of severe ADR, especially focusing on genetic variants of human leukocyte antigens (HLA), T cell receptor (TCR), drug-metabolizing enzymes, and drug-transporters (Figure 1). Up to present, the U.S. Food and Drug Administration (FDA) has labeled more than 180 approved drugs with genetic factors (Administration, 2021).

### Human Leukocyte Antigens

Type B idiosyncratic reactions is thought to be elicited by the excessive activation of CD4^+^ and CD8^+^ T-lymphocytes (Lerch and Pichler, 2004). Drugs or their reactive metabolites considered as foreign antigens that bind to receptors, activating the immune reactions. HLA are the primary immune anchors for presenting foreign antigens and responsible for pathogenesis of SCAR and DILI (Phillips et al., 2011; Chung et al., 2016a; Stephens et al., 2021). The highly polymorphic properties of HLA molecules among individuals provide diverse opportunities for interactions with various drugs. A specific type of HLA protein may have a higher affinity toward drug/metabolite antigens, presenting the antigen to TCRs, resulting in the activation of T lymphocytes, clonal expansion, skin inflammation, organ damage, and epidermal detachment.

The increasing data have been found a link between HLA alleles and severe ADR (Table 1 **)** in the last two decades. Carbamazepine (CBZ), belongs to aromatic and antiepileptic drug, is one of the common culprit drug(s) of SJS/TEN in different ethnic groups (Roujeau et al., 1995). HLA-B*15:02 is firstly reported to be strongly associated to carbamazepine (CBZ)-induced SJS/TEN in Chinese population (odds ratio [OR] = 2504) (Chung et al., 2004), and the association is latterly validated in different populations, such as Thai, Malaysian, Chinese, and Indian patients (Hung et al., 2006; Locharernkul et al., 2008; Mehta et al., 2009; Tassaneeyakul et al., 2010; Cheung et al., 2013; Tangamornsuksan et al., 2013; Chung et al., 2016b). Furthermore, it’s been proven that HLA-A*31:01 is associated with CBZ-induced hypersensitivity (Kim et al., 2011; McCormack et al., 2011; Ozeki et al., 2011), especially for DRESS patients (OR = 13.2) (Genin et al., 2014). Recently, HLA-B*57:01 is also identified to be associated with CBZ-induced SJS/TEN in Europeans (OR = 9.0) (Mockenhaupt et al., 2019). The phenotype-specific and ethnicity-specific are found in CBZ-induced SCAR patients. Oxcarbazepine (OXC) is another aromatic and antiepileptic drug that has a similar structure of carbamazepine, and HLA-B*15:02 allele is also found to be associated with OXC-induced SJS/TEN (OR = 27.9) (Chen et al., 2017). Furthermore, Asian patients carry the alleles of HLA-B*15:02, HLA-B*13:01, and HLA-B*51:01, have found a higher risk to induce phenytoin-induced SCAR (Chung et al., 2014; Su et al., 2019).

**TABLE 1:** Genetic associations with severe ADR in HLA, TCR, drug metabolism enzymes, and drug transporters.

Causative Drug	Genetic factor	Ethnicity	Sample size (case/ctrl)	OR	ADR	Ref.
Abacavir	HLA-B*57:01	Australian, American, Multiple Ethnicities	18/167	117 (29–481)	Hypersensitivity	Mallal et al. (2002)
			85/115	23.6 (8.0–70.0)		Hetherington et al. (2002)
			564/725	44.3 (24.5–80.3)		Sousa-Pinto et al. (2015)
Acetaminophen	HLA-A*02:06	Japanese	80/639	6.0 (3.7–9.9)	SJS/TEN	Ueta et al. (2019)
Allopurinol	HLA-B*58:01	Chinese, Thai, Korean, Japanese, European, Multiple Ethnicities	51/228	580.3 (34.3–9780.9)	SCAR	Hung et al. (2005)
			27/54	348.3 (19.2–6336.9)		Tassaneeyakul et al. (2009)
			25/57	97.7 (18.3–521.5)		Kang et al. (2011)
			58/493	40.83 (10.50–158.9)		Kaniwa et al. (2008)
			27/1822	80 (34–187)		Lonjou et al. (2008)
			164/8971	57.33 (35.09–93.67)		Ng et al. (2016)
				**SJS/TEN**		
Carbamazepine	HLA‐A*31:01	European, Japanese, Korean	22/3987	12.41 (1.27–121.03)	Hypersensitivity	McCormack et al. (2011)
			10/8862	49.9 (12.9–193.6)	DRESS	Mockenhaupt et al. (2019)
			77/420	9.5 (5.6–16.3)	SCAR	Ozeki et al. (2011)
			24/535	10.3 (4.4–24.2)	SCAR	Kim et al. (2011)
	HLA‐B*15:02	Chinese, Thai, Malaysian, Indian	60/144	1357 (193.4–8838.3)	SJS/TEN	Chung et al. (2004); Hung et al. (2006); Cheung et al. (2013)
			27/275	89.25 (19.25–413.83)		Locharernkul et al. (2008); Tassaneeyakul et al. (2010)
			6/50	25.5 (2.68–242.61)		Tangamornsuksan et al. (2013)
			42/42	54.76 (14.62–205.13)		Mehta et al. (2009)
			6/8	221.00 (3.85–12694.65)		
	HLA‐B*57:01	European	28/8862	9.0 (4.2–19.4)	SJS/TEN	Mockenhaupt et al. (2019)
	TCRβ CDR3 “ASSLAGELF”	Multiple Ethnicities	-	-	SJS/TEN	Pan et al. (2019)
Co-trimoxazole (Trimethoprim-sulfamethoxazole)	HLA-B*13:01	Chinese, Thai, Malaysian	41/138	45 (18.7–134)	DRESS	Wang et al. (2021)
			30/91	3.88 (1.56–9.63)		Sukasem et al., (2020); Wang et al. (2021)
						Wang et al. (2021)
	HLA-B*15:02,	Thai	30/91	3.47 (1.25–9.63)	SJS/TEN	Kongpan et al., (2015); Sukasem et al. (2020)
	HLA-C*08:01		43/91	3.91 (1.42–10.92)		
	HLA-B*38:02	Chinese, Thai	91/2545	2.5 (1.4–4.3)	SJS/TEN	Wang et al. (2021)
	HLA-B*38	European	25/1822	8.6 (3.5–21)	SJS/TEN	Lonjou et al. (2008)
	HLA-A*11:01	Japanese	15/2878	9.84 (3.35–28.9)	SCAR	Nakamura et al. (2020)
	HLA-B*14:01	European American	51/12156	9.20 (3.16–22.35)	DILI	Li et al. (2021)
	HLA-B*35:01	African American	10/5439	-	DILI	Li et al. (2021)
Dapsone	HLA-B*13:01	Chinese, Thai	7/677	49.64 (5.89–418.13)	DRESS	Wang et al. (2013); Zhang et al. (2013); Chen et al. (2018)
						Satapornpong et al. (2021)
			20/102	122.1 (23.5–636.2)		
			11/40	40.50 (6.38–257.03)		
Nevirapine	HLA-B*35:05	Thai, Indian	137/185	18.96 (4.87–73.44)	SJS/TEN	Chantarangsu et al. (2009)
			40/40	3.378 (1.541–7.405)		Umapathy et al. (2011)
Oxcarbazepine	HLA‐B*15:02	Chinese, Thai	20/-	27.90 (7.84–99.23)	SJS/TEN	Chen et al. (2017)
Penicillin	HLA-B*55:01	European	87996/1031087	1.30 (1.25–1.34)	Allergy	Krebs et al. (2020)
Phenytoin	HLA-B*15:02, HLA-B*13:01, HLA-B*51:01	East Asians (Chinese, Thai, Japanese)	15/275 (Chinese) 4/50 (Thai) 128/367 (Japanese, Taiwanese, Thai)	1.81 (0.85–3.85) HLA-B*13:01 18.5 (1.82–188.40) HLA-B*15:02 3.69 (1.91–7.11) HLA-B*51:01	SCAR	Locharernkul et al. (2008); Cheung et al. (2013); Chung et al., (2014); Su et al., (2019)
	HLA-B*15:13	Malaysian	13/300	8.56 (2.72–26.88) **SJS/TEN**	SCAR	Chang et al. (2017)
				50.73 (2.57–1002.07) **DRESS**		
	CYP2C9*3	East Asians (Chinese, Thai, Japanese)	105/3655	12 (6.6–20)	SCAR	Chung et al., (2014); Su et al., (2019)
Strontium ranelate	HLA-A*33:03	Chinese	8/8	25.97 (3.08–219.33)	SJS	Chen et al. (2021)
Vancomycin	HLA-A*32:01	European	19/46	403 (20.69–7849.44)	DRESS	Konvinse et al. (2019)
Amoxicillin-Clavulanate	HLA-DRB1*15:01	European	20/60	7.56 (2.85–20.03)	DILI	Hautekeete et al., (1999); Donaldson et al. (2010); Lucena et al., (2011)
			177/219	0.8 (0.1–5)		
			32/191	2.59 (1.44–4.68)		
Flucloxacillin	HLA-B*57:01	European	43/64	80.63 (22.81–284.96)	DILI	Daly et al. (2009)
Lumiracoxib	HLA-DRB1*15:01	Multiple Ethnicities	41/176	7.5 (5.0–11.3)	DILI	Singer et al. (2010)
Pazopanib	HLA-B*57:01	Asian, European	1188/1002	2 (1.3–3.1)	DILI	Xu et al. (2016)
Terbinafine	HLA-A*33:01	European, American	283/10588	2.7 (1.9–3.8)	DILI	Nicoletti et al. (2017)
Anti-tuberculosis drug	NAT2	Indonesian	50/191	4.75 (1.8–12.55)	DILI (non-allergic)	Yuliwulandari et al. (2016)
Clopidogrel	CYP2C19*2	European	-	2.42 (1.18–4.99)	Adverse cardiovascular symptoms	Miao et al. (2009); Shuldiner et al., (2009); Mega et al (2010)
Cyclosporine	ABCB1 (34355TT)	European	97/537	13.4 (1.2–148)	Nephrotoxicity	Hauser et al. (2005)
Sulfonylurea	CYP2C9*2 and *3	Multiple Ethnicities	759/2010	1.24 (1.03–1.48)	hypoglycemia	Yee et al. (2021)
Sulphonamides, anti-malarial drug, uricolytic agents	G6PD deficiency	Multiple Ethnicities	-	-	Hemolytic anemia	Beutler, (1991)
Irinotecan	UGT1A1*6 and *28	African, European	26/92	7.23 (2.52–22.3)	Neutropenia	Ando et al. (2000); Yang et al. (2018)
			791/6742	3.03 (2.05–4.47)		
Thiopurine	TPMT	European, American	398/679	2.3 (1.7–3.1)	leukopenia	Budhiraja and Popovtzer (2011); Avallone et al., (2014); Walker et al. (2019)
			98/1712	1649.69 (102.07–26662.44)		
	NUDT15 (*p*.Arg139Cys)	Asian (Chinese, Japanese Korean, and Indian)	47/45	7.20 (2.49–20.80)	leukopenia	Tanaka et al., (2015); Kakuta et al. (2016); Moriyama et al. (2016); Kim et al. (2017); Fei et al. (2018a); Fei et al. (2018b); Banerjee et al. (2020)
			34/135	212 (12.1–3737)		
			20/84	1.84 (3.98–36.02)		
Simvastatin	SLCO1B1 (rs4149056/rs4363657)	Multiple Ethnicities	32/16	4.5 (2.6–2.7)	Myopathy	Pasanen et al. (2006); Group et al. (2008)
Warfarin	CYP2C9*2 and *3	Multiple Ethnicities	3895/3896	0.35 (0.01–9.18)	Bleeding	Sridharan and Sivaramakrishnan, (2021)
	VKORC1	Multiple Ethnicities	3781/3783	0.93 (0.33–2.59)	Bleeding	Sridharan and Sivaramakrishnan, (2021)

Abbreviation: ABC, ATP-binding cassette; ADR, Adverse drug reaction; CDR3, complementarity determining region three; CYP, Cytochrome P450; DILI, Drug induced liver injury; DRESS, Drug reaction with eosinophilia and systemic symptoms; G6PD, Glucose-6-phosphate Dehydrogenase; NAT2, N-acetyltransferase two; NUDT15, Nudix hydrolase 15; HLA, Human leukocyte antigen; SCAR, Severe cutaneous adverse reactions; SLCO1B1, Solute carrier organic anion transporter family member 1B1; SCAR, severe cutaneous adverse drug reactions; SJS, Stevens-Johnson syndrome; TCR, T cell receptor; TPMT, thiopurine S-methyltransferase; TEN, Toxic epidermal necrolysis; UGT1A1, UDP Glucuronosyltransferase Family one Member A1; VKORC1, Vitamin K Epoxide Reductase Complex (VKORC).

Allopurinol is classified as a xanthine oxidase inhibitor and used to treat gout; however, it is known as one of the most common causes of SJS/TEN (Wang et al., 2019). Hung et al. have firstly identified that HLA-B*58:01 is strongly associated with allopurinol-induced SCAR in Chinese population (OR = 580.3) (Hung et al., 2005). This association was then verified in Japanese, South Korean, Thai, Hong Kong, European, Australia, and Portugal patients (Chung et al., 2007; Kaniwa et al., 2008; Lonjou et al., 2008; Tassaneeyakul et al., 2009; Kang et al., 2011; Lee et al., 2012; Ng et al., 2016).

Abacavir is effectively for treatment with HIV infection, and it has been reported that hypersensitivity reactions induced by abacavir is strongly associated with HLA-B*57:01 in Australia’s, U.S. and European populations (Hetherington et al., 2002; Mallal et al., 2002; Sousa-Pinto et al., 2015). In addition, HLA-A*02:06 is strongly associated with acetaminophen-related SJS/TEN with severe ocular complications in Japan population (Ueta et al., 2019).

HLA-B*13:01 has been recently reported to be associated with DRESS induced by sulfonamide, including dapsone (Wang et al., 2013; Zhang et al., 2013; Chen et al., 2018; Liu et al., 2019; Satapornpong et al., 2021), salazosulfapyridine (Yang et al., 2014), and co-trimoxazole (sulfamethoxazole-trimethoprim) (Wang et al., 2021) in Chinese or Thai populations, while HLA-A*11:01 is found to be associated with sulfonamide-related SCAR in Japanese population (Nakamura et al., 2020). The phenotype-specific is also observed in sulfonamide-induced ADR; for example, HLA-B*38:02 and HLA-B*15:02 was found to be associated with co-trimoxazole-induced SJS/TEN (Lonjou et al., 2008; Wang et al., 2021), but not with co-trimoxazole-induced DRESS.

Recently, Konvinse, et al. reported that HLA-A*32:01 is strongly associated with vancomycin-induced DRESS in a population of European ancestry (Konvinse et al., 2019), and the genome-wide association study (GWAS) conducted by Krebs et al. shows that HLA-B*55:01 is a genetic marker for penicillin allergy in United States, United Kingdom, and Estonian populations (OR = 1.4) (Krebs et al., 2020). Chen et al. further revealed that HLA-A*33:03 is associated with strontium ranelate-SJS (OR = 25.9) (Chen et al., 2021).

In addition to SCAR, several studies have identified the correlations between allergic DILI and specific HLA alleles. Amoxicillin-clavulanate (AC) is an antibiotic medication used to treat a variety of bacterial infections, but it is also considered as one of the most common culprit drugs of DILI (holding up to 10 ∼ 13% of DILI patients) (Andrade et al., 2005). The AC-induced DILI has been proved to be highly associated with HLA-DRB1*15:01 (Hautekeete et al., 1999). A GWAS study conducted by Lucena et al. has confirmed the HLA-DRB1*15:01 association and two novel HLA alleles associated with AC-induced DILI are further identified: HLA-A*02:01 in White European patients and HLA-B*18:01 in Spanish patients (Lucena et al., 2011). Both HLA class I and II alleles influence susceptibility to AC-induced DILI. Another common DILI inducing drug, lumiracoxib, is a COX-2 selective inhibitor nonsteroidal anti-inflammatory drug, like AC-induced DILI, has been identified that HLA-DRB1*15:01 is correlated with lumiracoxib-induced DILI (OR = 5.0) (Singer et al., 2010).

Flucloxacillin, belongs a narrow-spectrum beta-lactam antibiotic and used widely to treat patients with staphylococcal infections, is also a common cause of DILI. Daly et al. previously identified HLA-B*57:01 is strongly associated with flucloxacillin-induced DILI (OR = 80.6) (Daly et al., 2009). The same allele as HLA-B*57:01 is associated with pazopanib-induced DILI in Europeans (Xu et al., 2016). In fact, HLA-B*57:01 is also found to be strongly associated with abacavir hypersensitivity and CBZ-induced SJS/TEN in European descendants. These results suggest that HLA-B*57:01 is regarded as the most common risk allele for severe ADR, including SCAR and DILI, in European descendants.

Currently, Li et al. identified that HLA-B*14:01 allele is the highest associated HLA with co-trimoxazole (sulfamethoxazole-trimethoprim)-related DILI in European Americans (OR = 9.2), while HLA-B*35:01 is the most associated allele in African Americans (Li et al., 2021). In the recent research using the GWAS study, Nicoletti et al. discovered that HLA-A*33:01 is associated with DILI, especially with terbinafine-induced liver injury (OR = 40.5) (Nicoletti et al., 2017).

### T Cell Receptors

In addition to HLA alleles, several studies have shown that specific TCRs play important roles in the pathogenesis of severe ADR (Pirmohamed and Park, 2003; Pan et al., 2019). Pan et al. identified a public TCR composed of a TCRα complementarity determining region 3 (CDR3) “VFDNTDKLI” paired with a TCRβ CDR3 “ASSLAGELF” in clonotypes derived from patients of Asian and European descent with CBZ-induced SJS/TEN (Abel et al., 2008), which may explain how patients with different HLA alleles associated with different ethnicities can develop similar hypersensitivity reactions. This drug-specific TCR shows phenotype-specificity in an HLA-B*15:02-favored manner. In addition, Zhao et al. reported a promiscuous immune response associated with HLA Class-II‒-restricted T cells in patients with dapsone-induced DRESS (Zhao et al., 2021), but the detailed interactions and mechanisms that underlie HLA-B*13:01/dapsone–restricted CD8^+^ T cell responses remain poorly understood. The recent discovery of HLA genetic predispositions and oligoclonal and clonotype-specific TCR usages (Ko et al., 2011; Chung et al., 2015a) support the concept that an immune synapse involving an HLA–drug–TCR interaction is essential for inducing type B idiosyncratic ADR.

### Drug Metabolizing Enzymes

The gene polymorphism in drug metabolizing enzymes have also been attributed to ADR. Although previous studies shows that it have mainly been involved in dose-dependent mild ADR, a number of researches revealed that genetic defects of drug metabolizing enzymes also be responsible for the development of type B ADR (Pirmohamed and Park, 2003). The divergences in individual metabolism and drug clearance may contribute to occurrence and prognosis of ADR.

Cytochrome P450 (CYP) belongs to a superfamily of heme-containing enzymes responsible for oxidative biotransformation of a broad list of molecules (Kalgutkar et al., 2007). Modifications of its activity can be brought by the genetic polymorphisms, which may result in three phenotypes, such as poor, extensive, and ultra-rapid metabolizers (Sikka et al., 2005). There are at least 57 human genes known to code for CYP enzymes. CYP2D6, CYP2C9 and CYP2C19 genes were found to be responsible in 40% of biotransformation of drug, however, they were also regarded as one of the major susceptibility factors for ADR (Nebert and Russell, 2002; Zhou et al., 2009).

CYP2D6 accounts for the metabolism of 25% of drugs, and its polymorphism is highly relevant in altered enzymatic activity and ADR (Zhou, 2009). CYP2D6*3, *4, *5 and *17 are associated with poor metabolizers, and gene duplication of more than two normally-functioning alleles with ultra-rapid metabolizers (Zhou et al., 2009). Its substrates are mostly lipophilic and include antiarrhythmics, antipsychotics, antidepressants, opioids and some beta-blockers (Gardiner and Begg, 2006). One meta-analysis recommended reducing 50% of tricyclic antidepressant dose in patients who are CYP2D6 poor metabolizers (CYP2D6*4/*4 carriers) (Kirchheiner et al., 2004). Likewise, ultra-rapid metabolizers taking codeine may increase its active metabolite, morphine, resulting in life-threatening toxicity in patients taking the standard dose (Crews et al., 2012). Recently, a case report study identified two patients with CYP2D6*4 variant may be involved in severe ADR induced by quetiapine (Stäuble et al., 2021).

CYP2C9 contributes to 15% of metabolizing activity to drugs (Daly et al., 2017). Its substrates include anticoagulants, sulfonylureas, and some nonsteroidal anti-inflammatory drugs (Gardiner and Begg, 2006). *CYP2C9* genotype is an important predictor of warfarin-induced bleeding. In a meta-analysis study, patients with CYP2C9*2 and CYP2C9*3 alleles are poor metabolizers who are at a greater risk of bleeding, requiring lower doses of warfarin (Sanderson et al., 2005). Further studies showed that the shorter time to achieve therapeutic international normalized ratio (INR) for warfarin is observed in patients with both CYP2C9*2 and *3 and vitamin K epoxide reductase complex (*VKORC1C1173T*) genes (Sridharan and Sivaramakrishnan, 2021). CYP2C9 was also responsible for metabolism of phenytoin. CYP2C9*3 can reduce the clearance of phenytoin and has been found to be associated with development of phenytoin-induced SCAR (Chung et al., 2014). In addition, CYP2C9*2 and *3 alleles are found to enhance hypoglycemic effect in patients treated with sulfonylureas (Yee et al., 2021).

CYP2C19 metabolizes anti-depressants and proton pump inhibitors. Clopidogrel was metabolized into its active substance by CYP2C19. Loss of function in CYP2C19*2 and *3 alleles was associated with decrease in efficacy leading to increased ischemic complications (Miao et al., 2009; Shuldiner et al., 2009; Mega et al., 2010; Paré et al., 2010). Furthermore, a meta-analysis study demonstrated that poor metabolizers with CYP2C19 polymorphisms (CYP2C19*1, *2, and *17) are associated with increased risks in neurological, sexual and gastrointestinal side effects in patients taking citalopram/escitalopram (Fabbri et al., 2018).

Glucose-6-phosphate dehydrogenase (G6PD) is an important enzyme involved in red blood cell (RBC) oxidation through pentose phosphate pathway. Patients with *G6PD* deficiency are at a risk of hemolytic anemia after treatment with sulphonamides, anti-malarial drugs and uricolytic agents (Beutler, 1991). *G6PD* deficiency has also been reported to involve in primaquine- and dapsone-induced acute hemolytic anemia (Luzzatto and Seneca, 2014).

The genetic polymorphism of uridine diphospho glucuronosyltransferase 1A1 (UGT1A1*28) has been reported to reduce the UGT1A1 enzymatic activity and result in irinotecan-induced neutropenia (Ando et al., 2000). Further analysis study shows that Asians with the higher presence of UGT1A1*28 are more at a risk in developing irinotecan-induced toxicity compared to Western populations. Also, patients carried UGT1A1*6 are likely to develop irinotecan-induced toxicity (Yang et al., 2018).

N-acetyl transferase 2 (NAT2) is an acetylator enzyme found in the liver and gastrointestinal tract that reacts with drugs like dapsone, isoniazid, hydralazine, and sulfonamindes (Sim et al., 2014). Studies regarding its polymorphisms are responsible for its slow acetylator phenotype. It has been reported that patients with slow phenotype of NAT2 are associated with anti-tuberculosis nonallergic drug-induced liver injury (Yuliwulandari et al., 2016).

Thiopurine-induced leukopenia has been found to be associated with polymorphisms in thiopurine S-methyltransferase (TPMT) and Nudix Hydrolase 15 (NUDT15) genes, which encode TPMT and nudix hydrolase enzyme, respectively. Both enzymes are involved in thiopurine-containing drug metabolism such as azathioprine (Eichelbaum et al., 2006; Yang et al., 2015a). In meta-analysis studies, TPMT*3C variant is known to be associated with an increased risk in thiopurine-induced leukopenia in European descendants (Budhiraja and Popovtzer, 2011; Avallone et al., 2014; Walker et al., 2019). On the other hand, NUDT15 R139C (rs116855232, NUDT15*3) variant carriers are strongly associated with thiopurine-induced leukopenia in Asian populations, including Chinese, Japanese, Korean, and Indian populations (Tanaka et al., 2015; Kakuta et al., 2016; Moriyama et al., 2016; Kim et al., 2017; Fei et al., 2018a; Fei et al., 2018b; Banerjee et al., 2020).

### Drug Transporters

Drug transporters, responsible for influx and efflux of drugs, are categorized into two superfamilies: ATP-binding cassette (ABC) family, and solute carrier (SLC) family (International Transporter et al., 2010). Studies of correlation between drug transporter genes and ADR have increased noticeably. Associations of polymorphisms in *ABCB1* gene with cyclosporine-induced nephrotoxicity have been identified (Hauser et al., 2005). *ABCB1* also involved in ADR of osmotic-release oral system methylphenidate in adolescents (Kim et al., 2013). Furthermore, a meta-analysis study shows that patients carried ABCC2 3972T > T and ABCG2 34G > A genes are at a higher risk of irinotecan-induced neutropenia and diarrhea, respectively (Zaïr and Singer, 2016).

On the other hand, SLC drug transporter family has a well-known association with statin-related ADR (Niemi et al., 2006; Pasanen et al., 2006). Evidence revealed that the presence of C allele of rs4149056 and homozygous CC of rs4363657 of *SLCO1B1* show an increased risk to develop statin-induced myopathy (König et al., 2006; Group et al., 2008). Further study reported a significant association between patients carried SLCO1B1 T521C and myopathy induced by statins, including simvastatin, rosuvastatin and ceruvastatin (Xiang et al., 2018; Carr et al., 2019; Turner et al., 2020). It has also been reported that SLC6A3 rs28363170 is associated with haloperidol-related ADR (Zastrozhin et al., 2017), SLC22A2 rs316019 is associated with cisplatin-induced ototoxicity in cancer patients (Langer et al., 2020), and S allele of *SLC6A4* is involved in serotonin inhibitors-induced mania and gastrointestinal ADR (Zhu et al., 2017).

### Non-Genetic Risk Factors of Severe Adverse Drug Reactions

Patients with chronic kidney disease (CKD) and renal impairment may significantly delay drug clearance and metabolism, resulting in an increased risk of allopurinol-SCAR development and poor prognosis (Chung et al., 2015b), Furthermore, increased risks of allopurinol hypersensitivity have been significantly associated with female sex, CKD, cardiovascular disease (CVD) (Carnovale et al., 2014), allopurinol use starting after 60 years of age, and an initial dosage >100 mg/day. Allopurinol-associated mortality has found to be higher in patients with CKD, CVD, and older age (Yang et al., 2015b). Allopurinol prescribed for patients with asymptomatic hyperuricemia with underlying CKD or CVD also show an increased risk of hypersensitivity reactions and mortality (Yang et al., 2015b).

### Implementation of Pharmacogenomic Testing in Clinical Practice

Genetic HLA patterns associated with SCAR and DILI development have been identified for many drugs, and several pharmacogenetic markers have been successfully applied in clinical practice. Cost-effectiveness studies have examined the application of genetic testing before drug treatment to prevent SCAR development (Hughes et al., 2004; Ke et al., 2017; Plumpton et al., 2017), indicating that genetic screening is an important severe ADR prevention strategy. In fact, there are four prospective clinical trials have been conducted worldwide to demonstrate the clinical utility of HLA tests (including HLA-A*31:01, B*15:02, B*57:01, and B*58:01 genetic screening) (Mallal et al., 2008; Chen et al., 2011; Amstutz et al., 2014; University, 2017; Ke et al., 2019).

So far, a preventive genetic test for HLA-B*15:02 among potential new users of CBZ is supported by the national health insurance programs in Taiwan, Singapore, Hong Kong, Thailand, and mainland China (Chen et al., 2011; Tiamkao et al., 2013; Chen et al., 2014). The U.S. FDA further recommend genetic HLA-A*31:01 screening prior to the use of CBZ, and genetic HLA-B*15:02 screening before oxcarbazepine treatment, especially with ethnicities with high probability of HLA-B*15:02, such as Chinese and Thai. Recently, a trial is ongoing involving screening HLA to reduce ADR. (Identifier: NCT03184597).

Genetic HLA-B*57:01 testing prior to abacavir treatment for HIV treatment is widely used in clinical practice (Mallal et al., 2008) and is recommended by the U.S. FDA, European Medicines Agency, and Canada Health. However, HLA-B*57:01 genetic screening did not present a good result for new users before flucloxacillin treatment due to its low positive predictive value with 0.12% (17, 67). And, another HLA allele, HLA-B*57:03, is also found to be associated with DILI induced by flucloxacillin (141).

HLA-B*58:01 screening is commonly employed to protect patients from the risk of allopurinol-induced SCAR (Khanna et al., 2012). The American College of Rheumatology guidelines for the management of gout has recommended genetic HLA-B*58:01 testing prior to allopurinol use since 2012 (Khanna et al., 2012). Several medical centers in Hong Kong, Thailand, Korea, Taiwan, and mainland China provide such pre-screening (Ke et al., 2019). Furthermore, HLA-B*13:01 testing is recommended for new patients with leprosy being initiated on dapsone therapy in China (Liu et al., 2019); an ongoing clinical trial is examining the efficacy of CYP2C9*3 and *HLA-B* alleles screening to prevention of phenytoin-induced SCAR in China population (Chang et al., 2020).

The U.S. FDA has recommended genetic testing of *TPMT* and *NUDT15* polymorphisms prior to the use of thiopurine, especially for azathioprine. The British Society of Rheumatology guidelines have recommended that *TPMT* testing prior to prescribing azathioprine in Europeans (Chakravarty et al., 2008). As genetic *NUDT15* has shown to be strongly associated with thiopurine-related leukopenia in Asian populations, the preventive test of *NUDT15* for azathioprine has recently discussed to support by the national health insurance in China and Taiwan, but it still not approved.

### Current Trends and Future Perspectives

With the current available literature, there is an expanding number of published papers regarding genetic polymorphisms associated with severe ADR. Recently, the high-throughput technologies, such as whole genome sequencing (WGS) and whole exome sequencing (WES), have provided a rapid method to screen the genetic variants for patient and transformed the landscape of genetic biomarkers research. The use of pharmacogenetic testing, both reactively and preemptively, have been successful in terms of response to treatment. Studies have showed that reactive testing could explain or predict the treatment outcome during drug administration, while preemptive testing can prevent severe ADR that may occur. A number of studies have supported the use of pharmacogenetic testing in terms of cost-effectiveness. These studies have shown that testing lessens the cost compared to the addressing the life-threatening severe ADR developed. To achieve success of its use, standard implementation process of pharmacogenetic testing should be taken in place. The knowledge and expertise of the people involved, strong financial support, integrated data systems and holistic team approach will be deemed necessary. It is more necessary to promote the education of genetic testing for physicians in district hospital and community clinics. Pharmacogenetic testing will become a cornerstone to the concept of personalized or precision medicine.
